# Dosimetric characteristics of the small diameter BrainLab™ cones used for stereotactic radiosurgery

**DOI:** 10.1120/jacmp.v13i1.3610

**Published:** 2012-01-05

**Authors:** Gocha Khelashvili, James Chu, Aidnag Diaz, Julius Turian

**Affiliations:** ^1^ Radiation Oncology Center Northwestern Memorial Hospital Chicago Illinois 60611 USA; ^2^ Department of Radiation Oncology Rush University Medical Center Chicago Illinois 60612 USA

**Keywords:** small field dosimetry, stereotactic radiosurgery, SRS cones

## Abstract

The purpose was to study the dosimetric characteristics of the small diameter (≤10.0 mm) BrainLAB cones used for stereotactic radiosurgery (SRS) treatments in conjunction with a Varian Trilogy accelerator. Required accuracy and precision in dose delivery during SRS can be achieved only when the geometric and dosimetric characteristics of the small radiation fields is completely understood. Although a number of investigators have published the dosimetric characteristics of SRS cones, to our knowledge, there is no generally accepted value for the relative output factor (ROF) for the 5.0 mm diameter cone. Therefore, we have investigated the dosimetric properties of the small (≤10.0 mm) diameter BrainLAB SRS cones used in conjunction with the iPlan TPS and a Trilogy linear accelerator with a SRS beam mode. Percentage depth dose (PDD), off‐axis ratios (OAR), and ROF were measured using a SRS diode and verified with Monte Carlo (MC) simulations. The dependence of ROF on detector material response was studied. The dependence of PDD, OAR, and ROF on the alignment of the beam CAX with the detector motion line was also investigated using MC simulations. An agreement of 1% and 1 mm was observed between measurements and MC for PDD and OAR. The calculated ROF for the 5.0 mm diameter cone was 0.692±0.008 — in good agreement with the measured value of 0.683±0.007 after the diode response was corrected. Simulations of the misalignment between the beam axis and detector motion axis for angles between 0.5°–1.0° have shown a deviation > 2% in PDD beyond a certain depth. We have also provided a full set of dosimetric data for BrainLAB SRS cones. Monte Carlo calculated ROF values for cones with diameters less than 10.0 mm agrees with measured values to within 1.8%. Care should be exercised when measuring PDD and OAR for small cones. We recommend the use of MC to confirm the measurement under these conditions.

PACS numbers: 87.53.Ly, 87.55.‐x, 87.53.Bn, 87.55.K‐

## I. INTRODUCTION

Stereotactic radiosurgery (SRS) differs from conventional radiation therapy in that: a) the volume of treated tissue is much smaller; b) the number of fractions is much smaller (it is 1 most of the time); and c) the dose per fraction is much larger. SRS requires either immobilization of the skull using stereotactic frames with localization of the target in the stereotactic coordinate system, or noninvasive immobilization in conjunction with image‐guided registration. It is routinely used to treat inoperable intracranial lesions such as acoustic tumors, pituitary adenomas, and brain metastases. In the past few years, an increase in utilization of SRS techniques has been reported for more benign and functional diseases.^(^ ^1^ ^–^ ^4^ ^)^ Some of these clinical sites (i.e., trigeminal neuralgia) require a single fraction of very large dose (80–90 Gy) delivered with a small diameter (4–5 mm) cone and spatial accuracy better than 1.0 mm. Although most clinical physicists have the tools and expertise to accurately measure and model the SRS cones with diameter >10.0 mm, there are a few challenges when the dosimetry of small cones (diameter <10.0 mm) is attempted. For example, accurate measurements of small field percent depth doses, beam profiles, and relative output factors (ROF) are hampered by a number of factors such as: detector characteristics,^(^ ^5^ ^)^ position uncertainties, beams steering stability,^(^ ^6^ ^)^ and lack of lateral scatter and electronic equilibrium,^(^ ^7^ ^)^ among others.

From the listed factors, the main source of uncertainties (other than drawbacks of particular detector systems) are volume averaging and exact positioning of the detector.^(^ ^8^ ^,^ ^9^ ^)^ The finite size of any detector results in underestimation of measured ROF^(^ ^5^ ^,^ ^7^ ^,^ ^10^ ^)^ which, in turn, may result in significant overdosage of the small radiosurgery PTV. To overcome the volume averaging effect, several different approaches have been suggested.^(^ ^5^ ^,^ ^7^ ^,^ ^8^ ^,^ ^10^ ^,^ ^11^ ^,^ ^12^–^14^ ^)^ The most straightforward and logical method is to employ a detector with a small active volume and high spatial resolution. Detectors in this category include microchambers, diodes, diamond detectors, gel dosimeters, and radiographic/radiochromic film.

Microchambers have problems related to their small but still finite size of active volume (with typical diameter of 1−5 mm) and nonwater equivalence of some of their components.^(^ ^5^ ^)^ In addition, microchambers display field‐size dependence and polarity effect arising from signals originating in chamber stems and cables.^(^ ^5^ ^)^

Diamond detectors are tissue‐equivalent and have a good spatial resolution. Although these properties would make them appropriate detectors for small field measurements, they are expensive and have been shown to have dose‐rate dependence.^(^ ^6^ ^)^ In addition, volume effect correction factors cannot be estimated properly due to the irregular shape of the active volume of diamond crystal.^(^ ^6^ ^)^

The use of unshielded diodes (SRS diodes) has been shown to be most promising.^(^ ^2^ ^)^ SRS diodes partially solve the detector volume averaging issue. But because of the small electron range in common diode material (such as silicon), they still represent intermediate‐size cavities for typical SRS fields. In addition, they introduce new issues that are associated with the energy, dose rate, and directional dependence of their responses. For correct interpretation of measurement results, modeling of Burlin's general cavity theory is required.^(^ ^2^ ^)^

Since none of the existing detectors are perfect for direct measurements in small fields, several methods have been suggested to correct the measured data. They include the deconvolution of detector size,^(^ ^7^ ^,^ ^15^ ^)^ deconvolution of beam size,^(^ ^16^ ^)^ and use of Monte Carlo simulations^(^ ^11^ ^,^ ^17^ ^)^ to determine dosimetric correction factors for small SRS fields. The detector size deconvolution methods are based on modeling of detector response function for specific ion chambers and extracting true dose distribution from measured dose profiles using modeled detector response kernel.^(^ ^7^ ^)^ The beam size deconvolution involves a modeling of a large field in terms of small Gaussian fields. The dose profiles are measured in large field using pinpoint ion chamber. Small field Gaussian profiles are extracted from measured large field data using the large field beam model.^(^ ^16^ ^)^ A series of attempts were successfully undertaken to try to produce commissioning data for small fields using Monte Carlo methods.^(^ ^11^ ^,^ ^12^ ^,^ ^16^ ^,^ ^17^ ^)^ These studies have shown that accurate modeling of the treatment unit could result in useful dosimetric data. This data can be used for input to the treatment planning systems, or as an independent verification of measured data using a variety of detectors.

One of the problems that we have encountered during commissioning of BrainLAB stereotactic cones of varying diameters was a virtual absence of published dosimetric data related to usage of a particular configuration. (BrainLab cones mounted on a Varian Trilogy linear accelerator (Varian Medical Systems, Palo Alto, CA) equipped with a SRS mode.) The goal of this study is to describe a simple methodology for obtaining a complete set of dosimetric data for the above‐mentioned configuration. The measurements were performed using widely available detectors, and the results were compared with Monte Carlo simulations. Special attention is directed to the output factors for which there are no widely accepted published values. We found that the dosimetric data is strongly dependent on particular geometry of cones and SRS flattening filter, and may not be useful for different accelerators with different cones of same diameters.

## II. MATERIALS AND METHODS

### A. Theory

The ROF for a cone with diameter A at a depth $d_{\mathrm{ref}}$ is defined as follows:

(1) $$ROF(A)=\frac{D_{w}(A,d_{ref}=15\;\text{mm})}{D_{w}(100\times 100\;{\text{mm}}^{2},\;d_{ref}=15\;\text{mm})}$$

where *w* indicates a measurement in water.

For small fields (such as created by SRS cones), the measurements of ROFs are subject to many uncertainties that in turn may lead to significant errors in dose calculations. The difficulties in the accurate measurements of ROF can be traced to three “equilibrium factors”:^(^ ^5^ ^,^ ^7^ ^)^ (a) the size of the detector used in the measurements, (b) the lateral electronic equilibrium (LEE) in the irradiated medium and detector material, and (c) the partial occlusion of the viewable part of the X‐ray source (focal spot on the target). Since there is no single detector that obeys all three equilibrium conditions simultaneously under small and reference field conditions, different detectors, such as diode for small fields and ion chamber for reference field, should be used. The measurements with two different detectors must be coupled through intermediate field size measurements for which both detectors would have a similar response. The optimal size of intermediate field is determined by estimating lateral electron range and selecting a field size to be a first cone diameter that exceeds estimated range. This LEE diameter $D_{\mathrm{LEE}}$ is accomplished according to the following “rule of thumb”:^(^ ^18^ ^)^

(2) $$D_{LEE}(g/{\text{cm}}^{2})=15.124\frac{\%dd(20)}{\%dd(10)}-10.086$$

where *%dd* (10) and %*dd* (20) are measured percentage depth dose at 10 and 20 cm depth for each cone. The comparison of approximate values of $D_{\mathrm{LEE}}$ with cone diameter is summarized in the Table 1. It shows that the 20 mm diameter cone is an appropriate choice for intermediate measurement.

**Table 1:** Lateral electron equilibrium (LEE) diameter vs. SRS cone diameter.

Cone (mm)	5	7.5	10	12.5	15	17.5	20	25	30
$D_{\text{LEE}}(\text{mm})$	16.5	16.9	17.6	18.4	18.5	18.6	18.7	19.2	19.7

Thus Eq. (1) should be modified as follows:

(3) $$ROF(A)=\frac{D_{w}(A)}{D_{w}(100\times 100\;{\text{mm}}^{2})}=\frac{D_{w}(A)}{D_{w}(20\;\text{mm})}\times \frac{D_{w}(20\;\text{mm})}{D_{w}(100\times 100\;{\text{mm}}^{2})}$$

It is suggested here that the first fraction in Eq. (3) be determined using an unshielded SRS diode or a similar detector. In this study, we selected the Scanditronix unshielded SRS diode (IBA Dosimetry America, Bartlett, TN USA). This diode detector is one of the smallest sized detectors available and has a sensitive volume of the detector material (Si) of only $0.017\;{\text{mm}}^{3}$. The Si diode responds differently to radiation compared to water.(12–13) This effect is especially pronounced for small fields where LEE is absent. In order to replace the ratio of signals collected in water by the ratio of signals collected in Si, a field size dependent correction factor $k_{si}^{w}(A)$ has been introduced:

(4) $$\frac{D_{w}(A)}{D_{w}(20\;\text{mm})}=\frac{k_{Si}^{w}(A)D_{Si}(A)}{k_{Si}^{w}(20\;\text{mm})D_{Si}(20\;\text{mm})}=k_{20\;\text{mm}}^{A}\frac{D_{Si}(A)}{D_{Si}(20\;\text{mm})}$$

Final expression for ROF is as follows:

(5) $$ROF(A)=k_{20\;\text{mm}}^{A}\frac{D_{si}(A)}{D_{Si}(20\;\text{mm})}\times \frac{D_{w}^{IC}(20\;\text{mm})}{D_{w}^{IC}(100\times 100\;{\text{mm}}^{2})}$$

where the first fraction in Eq. (5) was measured using unshielded Si diode and the second fraction was measured using PTW Markus type parallel plate ion chamber (PTW, Freiburg, Germany). The calculation of the correction factors $k_{20\;\text{mm}}^{A}$ was performed using Monte Carlo simulations (as will be described in detail in the Monte Carlo calculation section below).

### B. Measurements

Dosimetric data collection was in accordance with iPlan version 3.1 requirements (BrainLAB, Feldkirchen, Germany). Percentage depth doses (PDD) were measured using the Scanditronix 3D dosimetry system at SSD=100 cm using Scanditronix unshielded SRS diode. Off‐axis ratios (OAR) were collected at SSD=92.5 cm, 7.5 cm depth, using SRS diode for 5 mm cone and Exradin A16 microchamber (Standard Imaging, Middletown, WI, USA). The ROFs for BrainLAB cones were measured using combination of unshielded SRS diode and PTW Markus type parallel plate ion chamber as described above.

We have also investigated the dependence of all of the above dosimetric parameters on the alignment of the beam CAX with the detector motion line. For this purpose we have simulated PDDs with gantry tilt of 0.0°, 0.2°, 0.5°, 0.7°, 0.9°, 1.0° angles (SSD=100 cm). This simulation can reproduce two possible setup errors: effect of gantry's digital reading deviation from real gantry angle and/or possible deviation of detector motion from vertical line during PDD scan performed during commissioning measurements.

### C. Monte Carlo simulations

**Figure 1:**
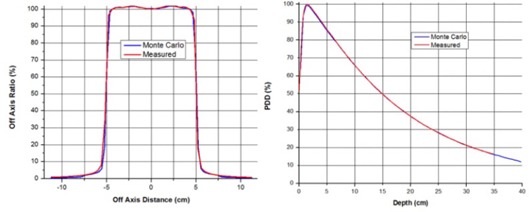
Results of Monte Carlo simulations vs. measurements for $10\times 10\;{\text{cm}}^{2}$ open field for Trilogy in SRS mode.

Monte Carlo simulations were performed in several steps. First we used EGSnrc/BEAMnrc [http://irs.inms.nrc.ca/software/egsnrc/] system to simulate the accelerator head to obtain phase space data for $100\times 100\;{\text{mm}}^{2}$ reference field size at SSD=100 cm. The Trilogy linear accelerator is equipped with a high output mode (6 MV‐SRS, 1000 MU/min) which requires a specific flattening filter (FF), and has a field size limited to $150\times 150\;{\text{mm}}^{2}$. (A technical drawing of the SRS FF was provided by Varian Medical Systems under a research collaboration agreement.) MC calculations were benchmarked against measured PDD and beam profiles (shown in Fig. 1).

Once the agreement between measured and calculated quantities was considered acceptable (1.0%, 1.0 mm), the SRS cones simulations were performed in stages. In the first stage, the accelerator head was simulated from the target to the Mylar exit window by setting the jaws to $50\times 50\;{\text{mm}}^{2}$, a configuration which is invariant for all stereotactic cones. Phase space files were generated at this location and used as an input to the second stage. In the second stage, the 5.0, 7.5, and 10.0 mm diameter cones were simulated. The geometry of each cone was measured using a feeler gauge, since no technical drawings were available. The assembly holding the cones and the cones themselves were simulated using BEAMnrc's flattening filter component module that allows for the use of the directional bremsstrahlung splitting (DBS) variance reduction method. The DBS method creates particles directed toward the region of interest, which was set 1.0 cm larger than the corresponding cone diameter to account correctly for scatter into the field. The $50\times 50\;{\text{mm}}^{2}$ phase space file was used as source for the second stage, and space phase files were generated for all three cones at SSD=100.0 cm from the original source (target). For each cone, the number of particles in the final phase space files exceeded $2.5\times 10^{6}$. The values for energy cutoff for photon (PCUT) and electron (ECUT) transport were set to 0.01 and 0.7 MeV, respectively. The phase space files generated during the second stage of simulations were used to calculate 3D dose distribution in water phantom using MCSIM user code.^(^ ^8^ ^)^

**Figure 2:**
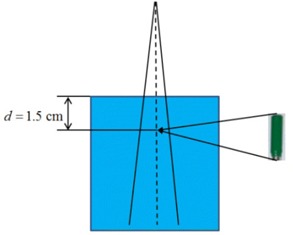
Monte Carlo simulation geometry for calculation of and factors.

The Scanditronix unshielded SRS diode was simulated according to the manufacturer specifications as a Si chip of area $0.6\;{\text{mm}}^{2}$ and thickness of 0.06 mm. Other components of diode, such as plastic cover, have not been used in the simulations. The water to silicon correction factor $k_{20\;\text{mm}}^{A}$ was calculated as follows. 3D dose calculations were performed using cones with diameters 5, 7.5, 10, 12.5, 15, 17.5, and 20 mm and the SRS diode inserted at d=1.5 cm depth in water using the geometry presented in Fig. 2. Similar calculations were performed in water without the presence of the diode. The central axis dose values at d=1.5 cm were extracted from both types of simulations (with and without Si) and $k_{Si}^{w}(A)=D_{w}/D_{Si}$ factors were determined for each of the cones. The factors $k_{20\;\text{mm}}^{A}$ were calculated by normalizing each of $k_{Si}^{w}(A)$ factors to $k_{Si}^{w}(20\;\text{mm})$.

## III. RESULTS

### A. Dose distributions

**Figure 3:**
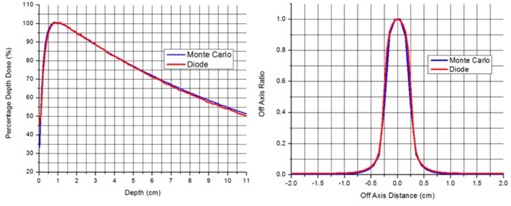
Monte Carlo simulations vs. measurements for 5 mm diameter cone for Trilogy in SRS mode.

Comparison of measured and calculated PDD and beam profiles for 5 mm cone are given in the Fig. 3. Agreement between measured values and Monte Carlo simulations is not surprising and was reported previously.^(^ ^10^ ^,^ ^11^ ^,^ ^17^ ^)^

**Figure 4:**
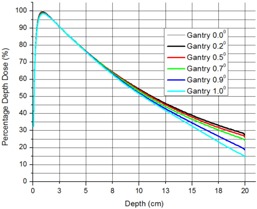
Dependence of PPD for 5 mm SDS cone on beam CAX alignment with detector line of motion.

**Figure 5:**
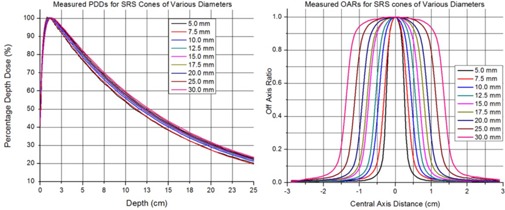
A complete set of PDDs and OARs for all cones.

Figure 4 shows the changes in MC calculated PDD for the 5.0 mm diameter cone as a function of gantry tilt with gantry angles indicated on the figure. The data show that deviations in PDD range from 0.5% to 15% at 15 cm depth. Figure 5 presents complete set of PDDs and OARs for all cones.

### B. Output factors

The summary of relative output factor calculations and measurements is given in the Table 2.

**Table 2:** ROF measured and calculated values ± 1 σ.

*Cone (mm)*	$k_{20\;mm}^{A}$	*ROF Meas.*	*ROF Corr.*	*ROF MC*	*Diode/MC*
5.0	0.961±0.011	0.711±0.007	0.683±0.011	0.692±0.011	1.32%
7.5	0.978±0.011	0.797±0.008	0.779±0.012	0.793±0.011	1.79%
10.0	0.983±0.011	0.850±0.008	0.836±0.012	0.850±0.011	1.67%
12.5	0.985±0.011	0.889±0.008	0.876±0.012	0.889±0.011	1.48%
15.0	0.991±0.011	0.905±0.009	0.897±0.013	0.906±0.011	1.00%
17.5	0.994±0.011	0.916±0.009	0.911±0.013	0.923±0.011	1.32%
20.0	1.000	0.926±0.009	0.926±0.009	0.933±0.011	0.75%

**Figure 6:**
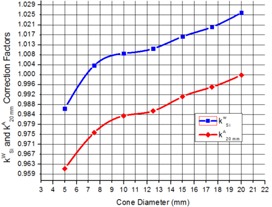
Calculated $k_{Si}^{w}(A)$ and $k_{20\times 20}^{A}$ factors.

**Figure 7:**
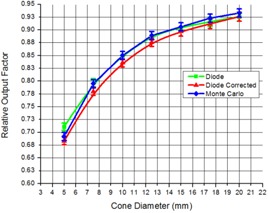
Relative output factors vs. cone diameter for Trilogy in SRS mode.

Column 2 of Table 2 represents calculated $k_{20\;\text{mm}}^{A}$ correction factors, and Fig. 6 represents correction factors $k_{Si}^{w}(A)$ and $k_{20\;\text{mm}}^{A}$ plotted versus cone diameter. The measured data before correction with $k_{20\;\text{mm}}^{A}$ factors is given in the third column of Table 2, and corrected and Monte Carlo‐calculated output factors are given in the fourth and fifth columns of Table 2, respectively. Figure 7 shows the measured, corrected, and Monte Carlo calculated ROFs versus SRS cone diameters. With all the corrections applied, the ROF value for the most controversial 5 mm diameter cone lies within the range from 0.676 to 0.690.

## IV. DISCUSSION

### A. Dose distributions

For measurements of PDDs and OAR there is no need of relating quantities measured under small field and reference geometry to each other. The Scanditronix unshielded SRS diode has proved to be a reliable detector for measuring PDD and OAR for SRS cones. These relative dosimetric quantities represent ratios of two measurements collected under the same lateral equilibrium conditions for each particular cone. Thus, agreement between measurements of PDDs and OARs and the Monte Carlo simulations for small cones is not surprising because they are determined independently from some “reference field”, as previously reported by several investigators.^(^ ^5^ ^,^ ^7^ ^,^ ^8^ ^,^ ^10^ ^,^ ^11^ ^,^ ^12^–^14^ ^)^ In regard to alignment of the beam CAX with the detector motion line measurements given in Fig. 4, the deviation between PDDs shows up beyond 7–8 cm depth. Since ROFs for cones are usually measured no deeper than 5.0 cm, the above misalignments will have negligible effect on their values. However, deviations during PDD measurements could have significant effects on the data entered in the treatment planning system and can lead to erroneous treatments.

### B. Output factors

Previously publishedROF data^(^ ^5^ ^,^ ^7^ ^,^ ^8^ ^,^ ^10^ ^,^ ^11^ ^,^ ^12^–^14^ ^,^ ^16^ ^,^ ^17^ ^)^ for radiation fields with 5 mm equivalent square sides ranges from 0.421–0.721. We must emphasize that in some of the above literature, radiation fields were created by MLCs, jaws, or cones of different variety. None of the data was collected for Trilogy linear accelerator in SRS mode with BrainLAB 5 mm cone configuration.

It was known that due to different equilibrium conditions in the small and reference fields, the measurement of ROF should be performed with combination of detectors (diode and ion chamber, for example). We found that the appropriate cone diameter that establishes a “link” between the diode and the ion chamber was the 20 mm cone (due to lateral equilibrium restoration). We used SRS diode up to 20 mm cone and Markus parallel plate ion chamber from 20 mm cone up to reference field size. We found that, in addition to linking diode and ion chamber detectors for different field sizes, an additional correction had to be made. This correction is related to the difference in response between the detector material (Si) and water. We found that the correction varies for different cones and it is up to 4% for the most controversial 5 mm cone. With this correction, the deviation between Monte Carlo calculated and measured output factors is no more than 1.8% for any cone, and is actually only 1.32% for the 5 mm cone.

It was previously reported^(^ ^12^ ^–^ ^13^ ^)^ that, for small fields, the x‐ray target is partially occluded by the field boundaries. In the above‐noted literature, the divergence of the small fields was such that the extrapolations of the openings towards the target converge slightly above the target position. As a result, the projection of the field opening at the position of the target is smaller than focal spot size, resulting in the partial occlusion of the X‐ray source. If part of the target is occluded, then geometrical penumbras from opposite edges of the fields are overlapping. This will result in a lower output when compared to the field sizes for which the entire focal spot on the target can be “viewed” by the detector. This suggests that occlusion effect is highly dependent on the design and divergence of the small field. We found that inner opening of the BrainLAB cones have negligible divergence, and the extrapolation of the opening backwards towards the target shows the inner opening of the cone converging at about 4 m above the target. At the level of the target, the projection of the field size exceeds the dimensions of the focal spot and, thus, virtually eliminates the occlusion effect.

## V. CONCLUSIONS

In this study, we have performed an experimental and theoretical investigation on the dosimetric parameters of the small SRS fields created using BrainLAB SRS cones in conjunction with a Trilogy accelerator equipped with a special SRS mode. The PDDs for various cones were measured using a Scanditronix unshielded SRS diode and were confirmed with Monte Carlo simulation. An agreement of 1% or better has been obtained. The OARs were measured using the same diode for 5.0 mm diameter cone and an Exradin A16 microchamber for larger cones. A similar agreement with Monte Carlo simulations has been reached.

The most important result of this work is the measurement and a detailed description of the method for obtaining reliable values for ROF. The method takes into account the nonequilibrium conditions presented in small radiotherapy fields, as well as the response difference between Si and water for different cone diameters. The ROF measured using the diode/Markus chamber combination and Eq. (3) for the 5 mm diameter cone is 711±0.007. After applying $k_{20\times 20}^{A}$ factor, the new value for this ROF is 0.683±0.011. Thus, corrected ROF values are within the uncertainty of the MC simulations. We found that the value for ROFs strongly depends on a particular configuration of accelerator and cones. Our result should not be taken as a general value for 6 MV beam with 5.0 mm cone configuration, instead it is specific to Trilogy‐type linear accelerators equipped with SRS mode (SRS flattening filter) and BrainLAB 5 mm SRS cone.

## ACKNOWLEDGMENTS

Authors would like to thank Varian Medical Systems and Dr. Jeffrey V. Siebers of Virginia Commonwealth University for providing us with linear accelerator treatment head geometry specifics. In addition, we would like to express our gratitude to Dr. C.‐M. Charlie Ma and Dr. Jinsheng Li of Fox Chase Cancer Center for providing us with MCSIM Monte Carlo user code.
